# Spatio-temporal variations of typhoid and paratyphoid fevers in Zhejiang Province, China from 2005 to 2015

**DOI:** 10.1038/s41598-017-05928-3

**Published:** 2017-07-18

**Authors:** Hua Gu, Wenjie Fan, Kui Liu, Shuwen Qin, Xiuyang Li, Jianmin Jiang, Enfu Chen, Yibiao Zhou, Qingwu Jiang

**Affiliations:** 1 Department of Epidemiology & Health Statistics, Fudan University, Shanghai, People’s Republic of China; 2 Department of Science Research and Information Management, Zhejiang Provincial Center for Disease Control and Prevention, Hangzhou, Zhejiang Province People’s Republic of China; 3 Department of Epidemiology & Health Statistics, Zhejiang University, Hangzhou, Zhejiang Province People’s Republic of China; 4 Department of Infectious Disease Control and Prevention, Zhejiang Provincial Center for Disease Control and Prevention, Hangzhou, Zhejiang Province People’s Republic of China

## Abstract

Typhoid and paratyphoid are two common enteric infectious diseases with serious gastrointestinal symptoms. Data was collected of the registered cases in Zhejiang Province from 2005 to 2015. The epidemiological characteristics were investigated and high-risk regions were detected with descriptive epidemiological methods and in-depth spatio-temporal statistics. A sharp decline in the incidences of both diseases was observed. The seasonal patterns were identified with typhoid and paratyphoid, one in summer from May to September was observed from 2005 to 2010 and the other lesser one in spring from January to March only observed from 2005 to 2007. The men were more susceptible and the adults aged 20 to 60 constituted the major infected population. The farmers were more likely to get infected, especially to typhoid. The Wilcoxon sum rank test proved that the incidences in the coastal counties were significantly higher than the inland. Besides, a positive autocorrelation was obtained with typhoid fever in global autocorrelation analysis but not with paratyphoid fever. Local autocorrelation analysis and spatio-temporal scan statistics revealed that high-risk clusters were located mainly in the coastal regions with typhoid fever but scattered across the province with paratyphoid fever. The spatial risks were evaluated quantitatively with hierarchical Bayesian models.

## Introduction

Typhoid and paratyphoid fevers, predominantly diseases of the reticuloendothelial system, are caused by *Salmonella* *enterica serovar Typhi* and *Salmonella* *enterica serovar Paratyphi* respectively and usually transmitted by fecal contamination of food or water through digestive system. These systemic infections may cause severe gastrointestinal problems, and their symptoms include persistent fever, diarrhea, hepatosplenomegaly and roseola^1^. They are still common around the world^2^ and their incidences vary in different countries and regions, depending on such factors as the infrastructure and medical treatment available and provided. The incidences were still substantially high in Africa and Asia, while sharp decreases have been observed in Europe and North America^3^. In China, the infections used to cause high morbidity and mortality up to the 1990s, particularly in such water-rich regions as Zhejiang Province^4, 5^. In recent years, the incidences have decreased thanks to the improved public sanitation and individual hygiene practices^6^ but fluctuations still exist.

Geographical information systems (GIS) and a wide array of spatial statistical methods have been employed for the surveillance of the communicable diseases and evaluation of the effectiveness of preventive interventions^7, 8^, presenting unique advantages over the conventional epidemiological techniques. For instance, an incidence map visualizes the diversity of typhoid and paratyphoid fevers in different regions. Besides, the in-depth statistics such as spatial autocorrelation analysis could demonstrate spatial distribution patterns and identify potential cluster areas^9, 10^. The spatio-temporal scan statistics can uncover the clusters in the dimension of space combined with time^11–13^, verifying the results of autocorrelation analysis and capturing the spatial and temporal details. The hierarchical Bayesian model has been employed to estimate spatial and temporal effects, offering a quantitative method to compare the effects among regions and years^14, 15^. All are helpful in identifying the high-risk regions, exploring the potential risk factors and evaluating the efficiency of existing targeted interventions.

Previous studies have reported that communicable diseases usually present obvious spatial and temporal patterns^16–20^. However, few studies have systematically investigated the epidemiological features and spatio-temporal distribution patterns of typhoid and paratyphoid fevers in Zhejiang Province, China. In this study, we aimed to give a full picture of epidemiology of typhoid and paratyphoid fevers, analyze the epidemiological characteristics and identify the regions with high risks spatially and temporally with the referred methods.

## Materials and Methods

### Study area

Zhejiang Province, economically developed, is located in the southeast China and borders the East China Sea, enjoying a subtropical monsoon climate^9^. The province, with 11 prefecture-level cities and 90 counties, has a land area of 101,800 square kilometers and a population of 55,030,001 by the end of 2015^21^.

### Data collection

All data, demographic and case-related, were obtained from China Information System of Disease Prevention and Control. The included cases were registered in the hospitals of the initial diagnosis in Zhejiang Province, distributed in 90 counties. Diagnosis and their confirmation were performed in accordance to the Diagnostic Criteria for Typhoid Fever and Paratyphoid Fever issued by Ministry of Health of the People’s Republic of China^22^. Specifically, the registered cases met the diagnostic criteria of clinical symptoms and laboratory evidence. All the confirmed cases had the clinical symptom of continuous fever of undetermined origin. Also, the serological specific antibody titer might increase to more than 4 times in acute phase than in the recovering phase; or *Salmonella* *enterica serovar Typhi* and *Salmonella* *enterica serovar Paratyphi* could be isolated from the blood, bone marrow, fecal or bile^22^. The laboratory reagents also met the national criteria.

Specifications extracted for each case included gender, age, address, occupation and date of onset. We used county as the basic unit for the spatial analysis and sorted the cases by county.

### Spatial autocorrelation, spatial stratified heterogeneity and spatial-temporal clusters (details in the Supplement of methods)

Autocorrelation between the adjacent regions was analyzed to identify distribution patterns. The general autocorrelation was employed to detect whether the epidemic was aggregated at the provincial level, and the local autocorrelation was employed to examine regional patterns and to ascertain the exact cluster locations^23–26^. Global Moran’s I and Local Moran’s I were utilized to fulfill such purposes^9, 23–26^. Besides, in this study, spatial stratified heterogeneity was also been explored to identify the possible variates caused by geographical and socio economic factors through the technology of Geodetector^27, 28^. Q-statistic in Geodetector was a powerful statistical indicator to measure spatial stratified heterogeneity, which has been widely applied in many natural and social researches^16, 29–31^. In this study, the location of bordering sea or not was detected to explore the potential heterogeneity.

Retrospective spatio-temporal permutation scan statistics were conducted to detect clusters during the study period. The spatio-temporal scan statistics are defined by a specific window with a circular geographic base, with height corresponding to time. The window size was constantly adjusted to detect possible spatial-temporal clusters^12, 13^. Log-likelihood Ratio (LLR) was employed to identify the special clusters by comparing the observed number with the expected one^32^. Monte Carlo test was conducted to determine the most likely clusters^13, 33^.

### Hierarchical Bayesian Model (details in the Supplement of methods)

Hierarchical Bayesian Model, complex but flexible, has been deemed as a powerful modality for robust estimation of the spatial-temporal effect proven by many epidemiologic studies^14, 34–36^. The prior information included in hierarchical model is generally the intrinsic constructional information and the inferred information of parameters, which can produce accurate and sound analysis, especially with large samples. Besides, the hierarchical method can simplify the explanation and calculation of the model and convenient Gibbs sampling^37–39, 40^.

Considering low incidences of both diseases, we assumed that the number of typhoid and paratyphoid cases registered yearly accorded with Poisson distribution as the first structure^14, 15^. Accordingly, a logit connection of relative risk was established to construct the second structure^14^. We finally constructed two models, Model One containing independent spatial and temporal effects and Model Two containing interactive spatio-temporal effect. Deviance information criterion (DIC) was adopted to determine the optimal fitness of both models, and Markov chain Monte Carlo (MCMC) algorithm to estimate parameters^41^. We ran the sample for 20000 iterations after the model was stable.

### Statistical software

The mapping and the autocorrelation analysis were conducted with ArcGIS software (version 10.1, ESRI Inc.; Redlands, CA, USA). The spatio-temporal clusters were detected with SatScan (version 9.1.1, Boston, MA, USA). The hierarchical model was constructed using WinBUGS (Version 1.4, Imperial College and MRC, UK) and GeoBUGS (Version 1.2, Imperial College and MRC, UK). Spatial stratified heterogeneity was analyzed with Q-statistic in Geodetector Software (http://www.geodetector.org/). Wilcoxon rank sum test, chi-square test and Gamma method of relevance analysis were conducted using SPSS (version 20, IBM Inc., Chicago, USA). All results were considered statistically significant if *P* < 0.05 for both sides.

### Ethics statement

This study was approved by the Ethics Committee of Zhejiang Provincial Center for Disease Control and Prevention. All personal information was kept confidential as required. All methods employed in this study were in accordance with the applicable guidelines and regulations.

## Results

### Epidemiological features

**Figure 1:**
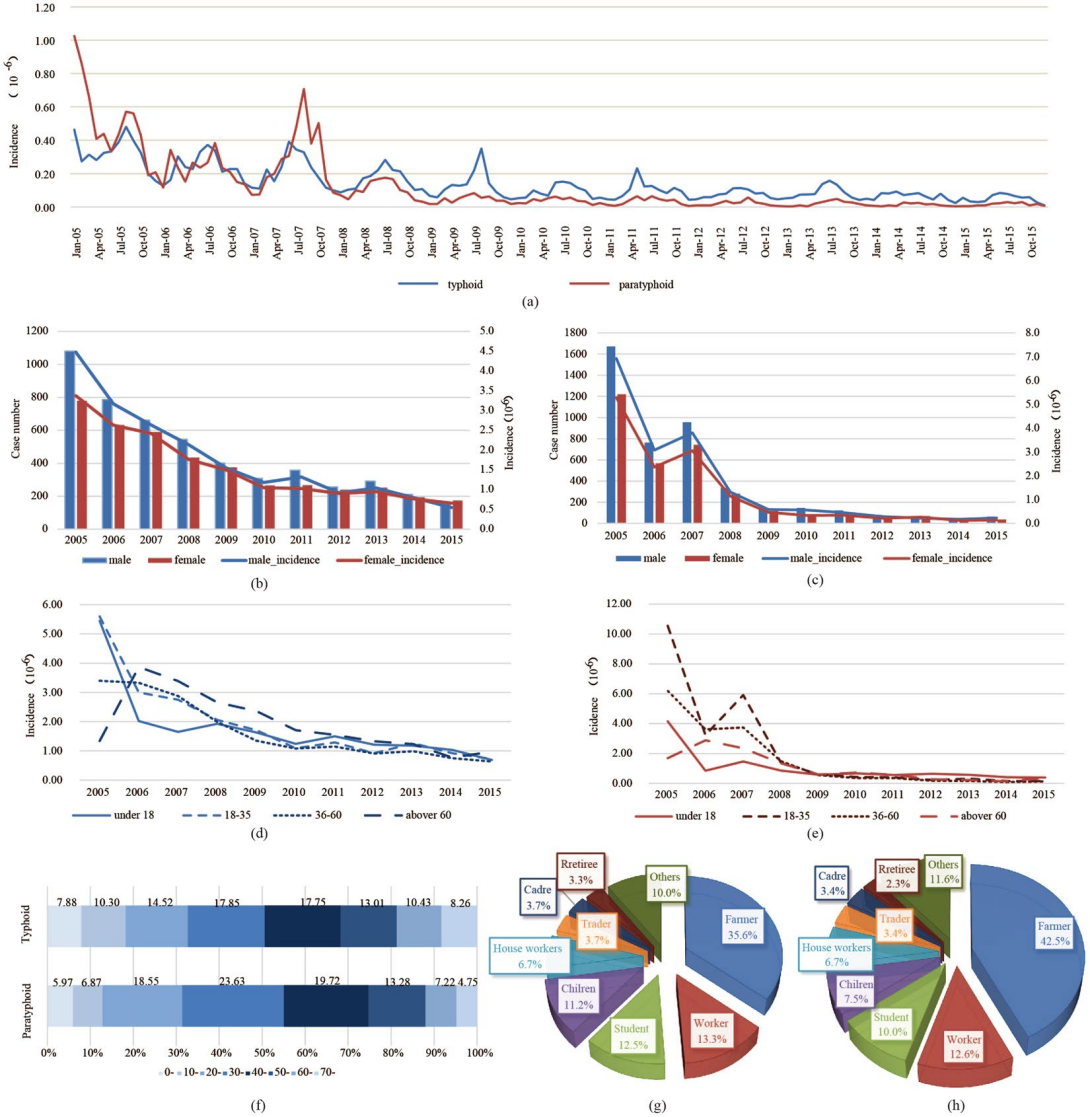
Epidemiological characteristics of typhoid and paratyphoid fevers in Zhejiang Province from 2005 to 2015. (**a**) Monthly distribution of typhoid and paratyphoid fevers (**b**) Gender distribution of typhoid fever (**c**) Gender distribution of paratyphoid fever (**d**) Age distribution of typhoid fever (**e**) Age distribution of paratyphoid fever (**g**) Age composition of typhoid and paratyphoid cases (**g**) Occupation distribution of typhoid fever (**h**) Occupation distribution of paratyphoid fever.

Included in our study were a total of 9264 typhoid cases and 7702 paratyphoid cases in Zhejiang Province from 2005 to 2015, those with unclear addresses having been excluded. A sharp decline in the incidences of both diseases was observed in the eleven years. The incidence of typhoid decreased from 3.94/10^6^ in 2005 to 0.60/10^6^ in 2015, and the incidence of paratyphoid decreased from 6.12/10^6^ in 2005 to 0.19/10^6^ in 2015. Two seasonal patterns were identified with typhoid and paratyphoid, one in summer from May to September and the other lesser one in spring from January to March. For typhoid, the summer peak were obvious from 2005 to 2010, and spring peak were obvious from 2005 to 2007. For paratyphoid, the summer peak were obvious from 2005 to 2008, and the spring peak were obvious in 2005 and 2006. Since then, the seasonality was getting weak gradually (Fig. 1a, Figs S4 and S5).

In terms of gender, both the numbers and the incidences of typhoid in the men surpassed the women every year (except 2015), the numbers and the incidences of paratyphoid in the men surpassed women every year (except 2013) (Fig. 1b and c). The Gamma analysis showed a relationship between age group and study year (γ = −0.120, *P* < 0.001). The proportions of groups aged from 18 to 35 and from 36 to 60 increased, while those under 18 and above 60 decreased. Additionally, the incidences of the group under 18 years old were stable during study period except in 2005, while other groups showed a trend of decline (Fig. 1d and e). And the results also showed that the majority of population was aged from 20 to 60, and these groups accounted about 60 percent both in typhoid and paratyphoid cases (Fig. 1f). As far as occupation is concerned, typhoid and paratyphoid fevers were most prevalent among the farmers and the difference in occupation had a statistical significance (*P* < 0.05) (Fig. 1g and h). Besides, the incidences of illnesses, especially those of typhoid among farmers surpassed those among the studied population as whole every year (Figure S1 in the Supplement).

### Incidence maps

**Figure 2:**
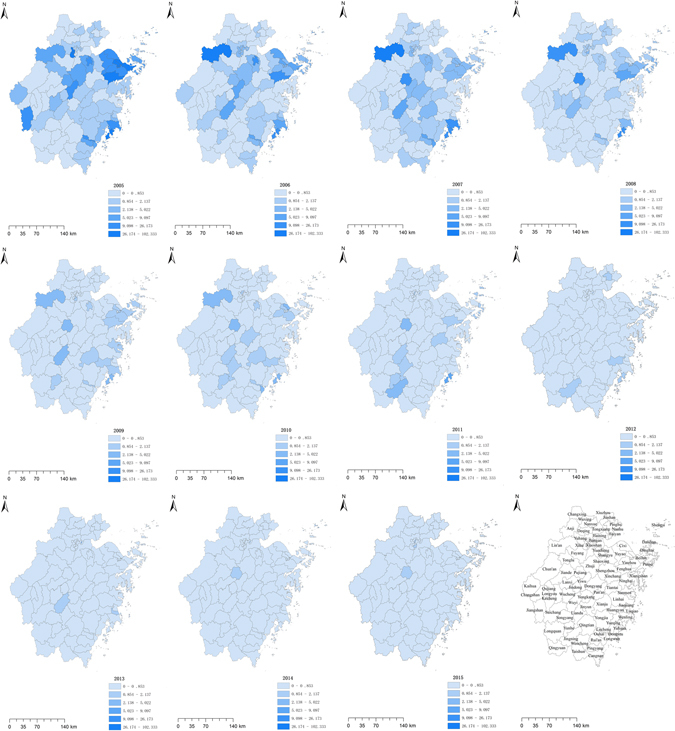
Maps of typhoid incidence for each county in Zhejiang Province from 2005 to 2015. These maps were created by ArcGIS software (version 10.1, ESRI Inc.; Redlands, CA, USA; homepage: https://www.esri.com/).

**Figure 3:**
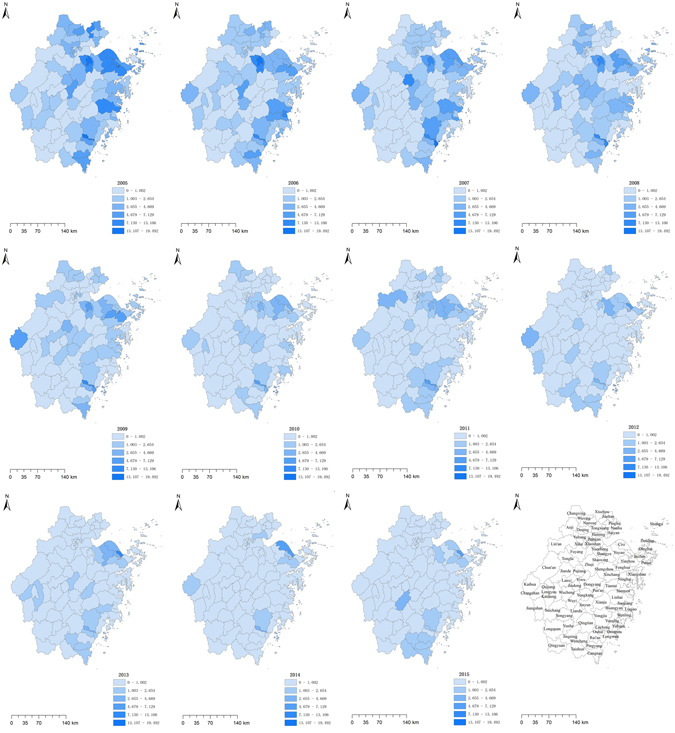
Maps of paratyphoid incidence for each county in Zhejiang Province from 2005 to 2015. These maps were created by ArcGIS software (version 10.1, ESRI Inc.; Redlands, CA, USA; homepage: ArcGIS software was https://www.esri.com.

The findings of the Wilcoxon rank sum test showed a spatial distribution with significantly higher typhoid incidences in the coastal cities than the inland ones (*P* < 0.05) on the prefecture level, but no such pattern was observed with the paratyphoid fever. On the county level, the typhoid and paratyphoid incidences of coastal counties were significantly higher than the inland ones (*P* < 0.05). The incidences of both illnesses were mapped in each county of the province from 2005 to 2015 (Figs 2 and 3). There were 39 counties with high typhoid incidence above 2.655 in 2005 while 2 counties in 2015, and 32 counties with high paratyphoid incidence above 2.138 while none in 2015. The maps showed high typhoid incidences in the coastal counties and high paratyphoid incidences in a number of scattered regions about from 2005 to 2009, and an obvious decline could be observed after 2010.

### Autocorrelation analysis and Spatial stratified heterogeneity

The global autocorrelation analysis for typhoid fever suggested a clustering distribution at the provincial level every year (Table 1). The local autocorrelation showed 98 high-high clusters in total with 3 high-low and 2 low-high clusters (Table S1 in the Supplement). High-high clusters were observed in the counties of Zhenhai (9 years), Cixi (9 years), Yuyao (8 years), Jiangbei (6 years), Yuecheng (7 years), Keqiao (6 years), Shangyu (6 years), Lucheng (10 years), Ouhai (7 years), Yongjia (6 years), and Longwan (5 years). These counties had high-high clusters for long periods. It is worth mentioning that 97 (94.17%) clusters were located in the municipalities of Ningbo, Shaoxing, and Wenzhou (Table S1 in the Supplement).

**Table 1:** Global autocorrelation analysis of typhoid and paratyphoid fevers in Zhejiang Province (●: Clustered, ○: Non-clustered).

Year	Typhoid Fever				Paratyphoid Fever			
	Moran’s I Index	Moran’s Z Score	Moran’s P Value	Mode	Moran’s I Index	Moran’s Z Score	Moran’s P Value	Mode
2005	0.660	9.664	<0. 001	●	0.781	10.073	<0. 001	●
2006	0.309	4.822	<0. 001	●	0.067	1.165	0.208	○
2007	0.259	3.889	<0. 001	●	0.016	0.666	0.486	○
2008	0.343	5.139	<0. 001	●	-0.025	-0.187	0.826	○
2009	0.324	4.806	<0. 001	●	0.066	1.087	0.261	○
2010	0.343	5.145	<0. 001	●	0.060	0.972	0.309	○
2011	0.310	4.542	<0. 001	●	0.008	0.297	0.752	○
2012	0.163	2.517	0.012	●	-0.020	-0.121	0.891	○
2013	0.420	6.546	<0. 001	●	0.612	9.191	<0. 001	●
2014	0.253	4.168	<0. 001	●	0.075	2.339	0.217	○
2015	0.238	3.728	<0. 001	●	0.581	8.548	<0. 001	●

The global autocorrelation analysis for paratyphoid fever showed significant positive autocorrelation only in the years of 2005, 2013 and 2015. The local autocorrelation detected a total of 32 high-high, 17 high-low, and 13 low-high clusters, with Yuhuan being deemed a high-risk region for 7 years (Table S2 in the Supplement).

Based on location of bordering sea or not, the spatial stratified heterogeneity was identified with the Q value of typhoid (0.115, *P* < 0.05) and paratyphoid (0.013, *P* < 0.05), implying location of bordering sea might be a possible influencing factor in our study.

### Spatio-temporal cluster analysis

The spatio-temporal cluster analysis detected 10 clusters of typhoid fever and 4 clusters of paratyphoid fever (Tables 2 and 3). The clusters were particularly obvious in the coastal regions in spring. For example, the most likely, the third and the fifth most likely clusters for typhoid fever were found in Ningbo (Haishu, Jiangdong, Jiangbei, Yinzhou, Fenghua, Ninghai, Xiangshan and Yuyao), Cixi and Shaoxing (Yuecheng, Keqiao), and the most likely cluster for paratyphoid was found in Ningbo (Haishu, Jiangdong, Jiangbei, Beilun, Zhenhai, Yinzhou, Yuyao and Fenghua) (details in Figures S2 and S3 in the Supplement).

**Table 2:** Spatio-temporal scan of typhoid fever in Zhejiang province from 2005 to 2015.

Cluster	Start Date	End Date	Districts	*P*	RR
Most Likely	2005/1/1	2005/2/26	Haishu, Jiangdong, Jiangbei, Yinzhou, Fenghua, Ninghai, Xiangshan, Yuyao	0.0000	3.9
2nd	2006/10/5	2006/12/4	Jiaojiang	0.0000	23.3
3rd	2014/2/3	2014/5/15	Cixi	0.0000	8.2
4th	2005/7/1	2005/11/18	Tiantai, Linhai	0.0000	3.2
5th	2006/2/15	2006/4/25	Yuecheng, Keqiao	0.0000	3.3
6th	2009/9/3	2013/6/29	Binjiang, Jinagan, Shangcheng, Xiaoshan	0.0000	2.3
7th	2007/9/27	2008/1/1	Pujiang	0.0000	8.7
8th	2015/5/26	2015/9/6	Wuyi	0.0000	17.8
9th	2009/7/14	2009/8/17	Lanxi	0.0013	18.3
10th	2010/7/15	2015/11/27	Ouhai, Lucheng, Yongjia, Wencheng, Ruian, Longwan, Liandu, Qingtian	0.0018	1.3

**Table 3:** Spatio-temporal scan of paratyphoid fever in Zhejiang Province from 2005 to 2015.

Cluster	Start Date	End Date	Districts	*P*	RR
Most Likely	2005/1/1	2005/3/12	Haishu, Jiangdong, Jiangbei, Beilun, Zhenhai, Yinzhou, Yuyao, Fenghua	0.0000	3.80
2nd	2007/6/27	2007/8/29	Lin’an	0.0000	5.36
3rd	2007/9/20	2007/10/23	Wenling	0.0000	7.41
4th	2010/8/15	2015/12/26	Shangcheng, Xiacheng, Jianggan, Gongshu, Xiaoshan, Yuhang, Nanhu, Xiuzhou, Jiashan, Haiyan, Haining, Pinghu, Tongxiang, Wuxing, Nanxun, Deqing	0.0000	3.46

### Hierarchical Bayesian model

**Figure 4:**
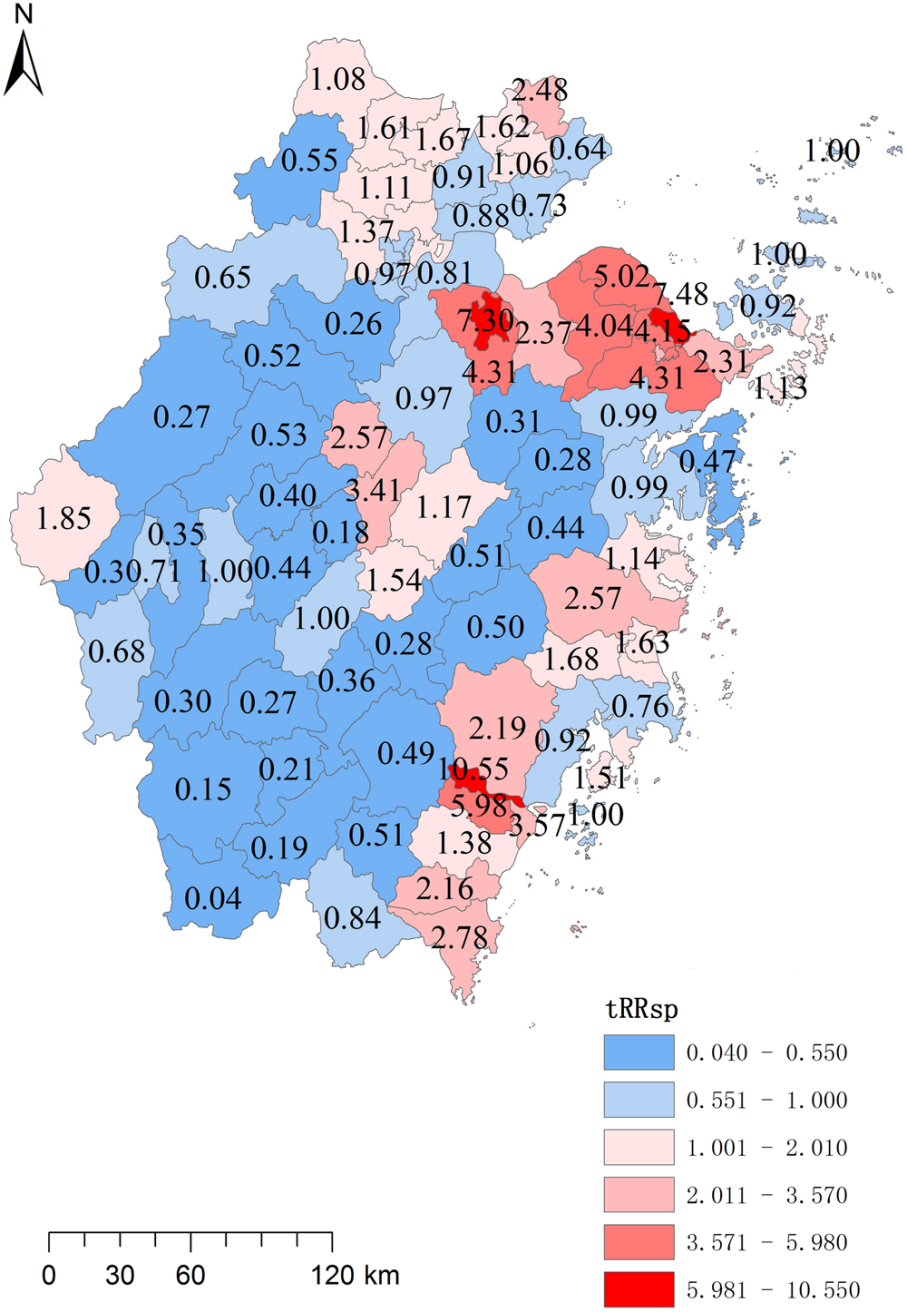
Independent spatial effect of typhoid fever for each county in Zhejiang Province from 2005 to 2015. This map was created by ArcGIS software (version 10.1, ESRI Inc.; Redlands, CA, USA; homepage: https://www.esri.com/).

**Figure 5:**
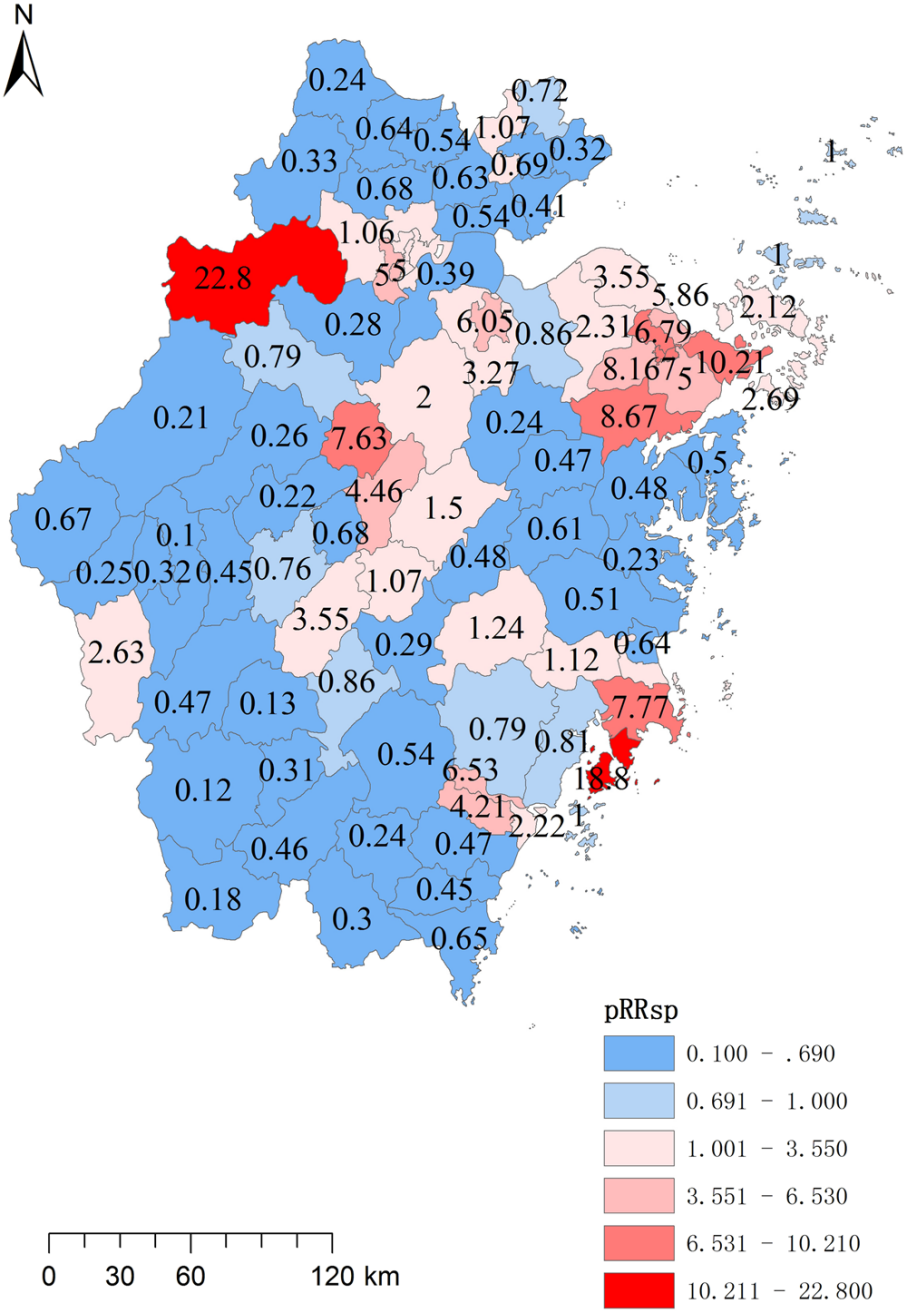
Independent spatial effect of paratyphoid fever of each county in Zhejiang Province from 2005 to 2015. This map was created by ArcGIS software (version 10.1, ESRI Inc.; Redlands, CA, USA; homepage: https://www.esri.com/).

The DIC value for typhoid fever was 1326.07 in Model One vs. 2306.25 in Model Two and that for paratyphoid fever was 2306.25 in Model One vs. 6244.34 in Model Two, suggesting that Model One boasted a better fitness for our data. Hierarchical Bayesian model was then used for both prefectures and counties with a view of identifying risk regions (Tables 4 and 5). Ningbo (RR = 2.51), Wenzhou (RR = 2.13) and Shaoxing (RR = 1.89) had high spatial risk effects for typhoid fever and Ningbo (RR = 2.85), Taizhou (RR = 2.25) and Hangzhou (RR = 1.90) had high spatial risk effects for paratyphoid fever. The temporal effects were high in the early years. The model constructed at the county level detailed spatial risk for each county (Figs 4 and 5).

**Table 4:** Spatial and temporal effect of typhoid fever for each prefecture each year.

Prefecture	Spatial effect Mean(95%CI)	Year	Temporal effect Mean(95%CI)
Hangzhou	0.62(0.67,0.72)	2005	1.53(1.62,1.71)
Ningbo	2.51(2.64,2.78)	2006	1.16(1.23,1.30)
Wenzhou	2.13(2.24,2.35)	2007	1.01(1.08,1.14)
Jiaxing	0.81(0.89,0.97)	2008	0.79(0.84,0.90)
Huzhou	0.88(0.97,1.07)	2009	0.61(0.66,0.71)
Shaoxing	1.89(2.01,2.13)	2010	0.45(0.49,0.53)
Jinhua	1.01(1.09,1.17)	2011	0.48(0.53,0.57)
Quzhou	0.57(0.64,0.72)	2012	0.38(0.42,0.46)
Zhoushan	0.72(0.85,1.00)	2013	0.41(0.45,0.49)
Taizhou	1.05(1.12,1.20)	2014	0.30(0.34,0.37)
Lishui	0.18(0.22,0.27)	2015	0.24(0.27,0.30)

**Table 5:** Spatial and temporal effect of paratyphoid fever for each prefecture each year.

prefecture	Spatial Effect Mean(95%CI)	Year	Temporal Effect Mean(95%CI)
Hangzhou	1.90(2.02,2.14)	2005	2.64(2.78,2.91)
Ningbo	2.85(3.01,3.19)	2006	1.19(1.27,1.35)
Wenzhou	0.87(0.94,1.01)	2007	1.52(1.61,1.70)
Jiaxing	0.36(0.42,0.48)	2008	0.53(0.58,0.63)
Huzhou	0.26(0.32,0.39)	2009	0.22(0.25,0.29)
Shaoxing	1.34(1.45,1.56)	2010	0.19(0.22,0.25)
Jinhua	1.37(1.48,1.59)	2011	0.17(0.20,0.22)
Quzhou	0.52(0.61,0.70)	2012	0.11(0.13,0.15)
Zhoushan	1.31(1.52,1.75)	2013	0.10(0.12,0.15)
Taizhou	2.25(2.39,2.53)	2014	0.06(0.07,0.09)
Lishui	0.23(0.28,0.34)	2015	0.08(0.09,0.11)

## Discussion

In this study, we explored the epidemiological features of typhoid and paratyphoid fevers, laying special emphasis on the detection of high-risk areas. In the descriptive analysis, we recorded the long-term trends, seasonal peaks, regional differences and the features in the respects gender, age and occupation.

An obvious decline of incidences was observed, suggesting that the prevention and control for typhoid and paratyphoid fevers had been effective to some extent. The paratyphoid incidences were close to those or sometimes surpassed the typhoid incidences from 2005 to 2007, after which typhoid fever was more prevalent than paratyphoid fever. Two temporal patterns of clusters were identified. The obvious temporal clusters fell during the summer months from May to September, which was in accordance with the seasonality of the enteric infectious diseases^42, 43^. In summer, bacteria grow and reproduce themselves vigorously thanks to the high temperature and humidity, and more infections may occur because people are more inclined to have cold dishes such as salad vegetables and uncooked seafood. As a coastal province, its residents consume various seafood, even uncooked, especially when people have gatherings. This dietary habits increased risk for the infection with typhoid and paratyphoid fevers because the marine products are the natural carriers of *Salmonella enterica serovar Typhi and Salmonella enterica serovar Paratyphi* ^43–46, 47^. With raw food or food not well cooked, the living pathogenic bacteria enter the body and cause diseases. This might account for the higher incidences in the coastal prefectures and counties. This summer pattern was also reported in other provinces^44^. Interestingly, a small peak related to Chinese Spring Festival from January to March was observed, when social activities usually surge, such as paying visits to relatives or friends and dining together^44, 45^. In addition, catering hygiene might hardly be guaranteed as a result of insanitary practices in the process of storage, preparation and cooking. For example, containers may be mixed up with the raw and the cooked food. Besides, a vast part of Zhejiang Province is still rural, featuring inadequate infrastructure to supply clean water and poor personal sanitary practices like using substandard food materials, adopting substandard processes, and living with insanitary dining styles. All these factors may lead to bacterial contamination of the food, probably causing typhoid and paratyphoid infections^48, 49^. However, this clustering pattern was obvious only from 2005 to 2007. Such an observation may have been due partially to the decreasing overall incidences and partially to the improved safety in water and food supply and enhanced public hygiene education. Besides, the seasonality of typhoid and paratyphoid was weak gradually after 2011, which might mirror the endeavors made for the improvement as changes in drinking water and lavatories in public health.

The result showed that the major workforce aged 20 to 60 years old constituted the major cases, for they had more chances to have contact with the patients and the carriers in daily life or on job and thus expose themselves to more risks. The farmers, the largest population in China, bear the heavy physical work. Besides farming, they take up odd jobs as construction workers and couriers. They usually do not enjoy adequate welfare and social security, and their health is subject to the poor working condition^43, 44, 45, 50^. The incidences among those under 18 years old were not much changed during study period in contrast to a sharp decrease among other age groups, suggesting that the interventions at school had not been effective. Outbreaks occasionally occur in the schools because of the low immunity of the children, dense population, crowded environment and insufficient health facilities particularly in some rural primary and middle schools^44^. This may serve as a reminder that more attention should be paid to the surveillance over and protection of these special population groups.

The incidence map showed high typhoid incidences in the coastal regions and some other regions, which agreed with the findings of the retrospective spatio-temporal scan analysis and hierarchical Bayesian model. One factor contributing to such high incidences might have been the dietary habits as mentioned above, and another lay in the fact that numerous immigrants under economic prosperity increased infectious disease mobility. The coastal areas, especially some counties under the municipality of Ningbo, Shaoxing, Taizhou, Wenzhou, and Yiwu, had attracted more migrant workers who might have been registered as farmer, and such population mobility facilitated the transmission of typhoid fever^45^. The third contributing factor for the high incidences might be abundant water resources in the north counties under municipality of Jiaxing, Huzhou and Hangzhou, all located around Taihu Lake, where the incidences and spatial risks were high especially in the early years before the 1990s^9^. Besides, other counties have particular causes for the high incidences. For instance, Kaihua is a mountainous area and also the headstream of Qiantang River. Its infrastructure, including water supply system, was out of date, and it is common that the residents there may drank water from springs or wells without sterilization and disinfection, resulting in high incidence.

Lin’an was a clustering area for paratyphoid fever with the highest spatial risk as indicated by the findings in the autocorrelation analysis, retrospective spatio-temporal scan and Bayesian model. The municipality was widely known for the paratyphoid infections from 2002 to 2007^51^, explained by the locals’ dependence on wells for fresh water which might have been affected by the polluted ground water. The spatial effect maps (Figs 4 and 5) showed a difference in high-risk regions between typhoid and paratyphoid fevers. For typhoid, high spatial risk was observed in almost all coastal areas and the counties around Taihu Lake. For paratyphoid, the coastal areas had two clusters, one in Ningbo and another bordering Wenzhou and Taizhou. High risks were also found in the middle counties of the province. In addition, the autocorrelation analysis and retrospective spatio-temporal scan statistic proved that typhoid fever had more obvious clusters than paratyphoid fever. This was echoed by Wilcoxon rank sum tests, which showed that the typhoid incidences in the coastal municipalities were significantly higher than those in the inland ones both on the prefecture level and on the county level but that paratyphoid incidences had significant difference only on county level, which was also verified by spatial stratified heterogeneity analysis of geographical location of bordering sea or not. We may conclude that typhoid and paratyphoid fevers had different clustering patterns and inferred that they had different risk factors. Factors specific of the coast areas such as seafood played a major role with typhoid fever, but other factors such as unclean water supply played an important role with paratyphoid fever^52^. In further studies, we aim to analyze the causes for clustering patterns by collecting data about risk factors and strain information of the two bacteria.

### Limitation

Several limitations should be mentioned in this study.

1. We excluded the cases registered with unclear addresses or with addresses in other provinces, but the infection usually occurred in Zhejiang Province. Such exclusion accounted for less than 5%, which exerted limited influence on the findings.
2. Chronic carriers and asymptomatic cases might not have gone to hospitals, and these group would not have been registered with the surveillance system. Thus, responder bias might be a critical confounding factor in this study.
3. With the limited demographic data, we were unable to show typhoid and paratyphoid incidences among occupational groups. For the same reason, the population were limited only to four age groups.
4. Local Moran’s I were able to demonstrate the epidemic pattern in spatial distribution but could be disturbed by population fluctuation;
5. In this study, the Q-statistic for spatial stratified heterogeneity was rough only based on the variable of bordering sea or not. More factors including meteorological, geographic, and socioeconomic information are needed for spatial stratified heterogeneity to make the result more accurate in the future.
6. When conducting the Bayesian model, we found the numbers of typhoid cases observed were inaccurate because they were calculated by rough age cohorts. The prior parameters chosen according to the literature might not be the most suitable^15^.
7. Due to inadequate meteorological, geographic, and socioeconomic information, the estimated independent spatial effect in Bayesian model remained to be explained. In further studies, we plan to put the risk factors in model as covariates.
8. More models such as spatial panel model might be employed to conduct temporal prediction and explain the spatial effect, and field investigation needs to be conducted to verify our findings.

## Conclusions

In this study, we investigated the epidemiological characteristics and identified spatial and temporal clusters of typhoid and paratyphoid fevers in Zhejiang Province from 2005 to 2015. Two seasonal clusters were detected: one was lesser in spring from 2005 to 2007 and the other was remarkable in summer from 2005 to 2010. The seasonality was weak gradually after 2010. The study revealed that the men and the adult aged 20–60 outnumbered other age groups. In relation to occupation, the farmers were the major population group of the infections. Geographically, the coastal counties had higher incidences of the typhoid and paratyphoid fevers than the inland. Clusters were found more obvious for typhoid fever than for paratyphoid fever. Typhoid fever clustered in the coastal regions, while paratyphoid fever clusters scattered across the province. The clustering areas were proved to have high spatial effect in the hierarchical Bayesian models. Despite its limitations, this study may contribute to the protection of high-risk population, the surveillance of high-risk regions, the formulation of interventions, and the management of water resources.

## Electronic supplementary material

Supplementary Information
