# Supplementary material for: Spatio-temporal variations of typhoid and paratyphoid fevers in Zhejiang Province, China from 2005 to 2015
Source: Sci Rep. 2017 Jul 18;7:5780. doi: 10.1038/s41598-017-05928-3 (PMC5515934; doi:10.1038/s41598-017-05928-3)
Supplement: Supplementary file 1 — Supplementary Information [file 41598_2017_5928_MOESM1_ESM.pdf]

Spatio-temporal variations of typhoid and paratyphoid fevers in Zhejiang Province, China from 2005 to 2015

Hua Gu[1,2], Wenjie Fan[3], Kui Liu[2], Shuwen Qin[4], Xiuyang Li[3], Jianmin Jiang[2], Enfu Chen[4], Yibiao Zhou[1*], Qingwu Jiang[1*]

Supplement of methods

1. Spatial autocorrelation

1.1 Global spatial autocorrelation

Autocorrelation representing the relationships of adjacent regions was analyzed to detect the distribution patterns. The global autocorrelation was to detect whether or not the epidemic was clustered at the provincial level. Two counties borders to each other will be defined they're adjacent. Moran's I was used mainly to estimate the independence or correlation of neighboring counties, with the coefficient calculated as follows:

$$I = \frac{n}{\sum_{i=1}^{n}\sum_{j=1}^{n} w_{ij}} \frac{\sum_{i=1}^{n}\sum_{j=1}^{n} w_{ij}(x_i - x)(x_j - x)}{\sum_{i=1}^{n}(x_i - x)^2}$$

where n is the number of counties; x is the average incidence in the counties; x_i and x_j are the incidences in counties i and j; w_{ij} is the matrix of spatial weights. If unit i was adjacent to unit j, $w_{ij} = 1$; otherwise, $w_{ij} = 0$. Moran's I Index ranges from -1 to 1. Moran's I > 0 implies clustering in the spatial distribution, Moran's Index < 0 dispersing in the spatial distribution, and Moran's Index = 0 a random spatial distribution. Z test is usually used as a hypothesis test to confirm a spatial clustering. If P <0.05, Moran's I ≠ 0 means that the area had clustering of typhoid infections.

1.2 Local spatial autocorrelation

As global spatial autocorrelation could not locate the exact clusters, local spatial autocorrelation was employed to investigate the regional patterns. Local Moran's I was calculated as follows:

$$I_i = \frac{n^2}{\sum_{i=1}^{n}\sum_{j=1}^{n} w_{ij}} \frac{w_{ij}(x_i - x)\sum_{j=1}^{n}(x_j - x)}{\sum_{j=1}^{n}(x_j - x)^2}$$

Besides, the result of local spatial autocorrelation showed significant region has four clustered types: high-high type(HH), high-low type(HL), low-high type(LH), low-low type(LL).

2. Spatial-temporal clusters

The retrospective spatio-temporal scan statistic based on permutation model was employed to detect the typhoid fever clusters during study period. The spatio-temporal scan statistic was defined with a window with a circular geographic base, with height corresponding to time. The base and the height of the windows were constantly adjusted to detect possible spatial-temporal clusters. The permutation model used in our study combine temporal and

spatial information. The permutation mode needed the data of cases, and adjacency and population data were not necessary. Log-likelihood Ratio (LLR) was employed to identify the special clusters by comparing the observed incidence with the expected one. Eventually, Monte Carlo test was conducted to determine the most likely clusters.

LLR was calculated as follows:

$$\mathrm{LLR} = \log\left(\frac{\mathrm{n}}{\mathrm{E(n)}}\right)^{\mathrm{n}}\left(\frac{\mathrm{N-n}}{\mathrm{N-E(n)}}\right)^{\mathrm{N-n}} \mathrm{I()}$$

where n is the number of cases in scanning window, N is the total number of cases in study area, $E(n)$ is the expected number of cases in scanning window, $\mathrm{I()}$ is the indicator function. If $n > E(n)$, $\mathrm{I()}$ equals to 1; otherwise, $\mathrm{I()}$ equals to 0.

The relative risk (RR) was calculated as follows:

$$\mathrm{RR} = \frac{{}^{\mathrm{n}}\!/_{\mathrm{E_{(n)}}}}{\mathrm{N-n}\Big/\left(\mathrm{E_{(N)}} - \mathrm{E_{(n)}}\right)}$$

3. Hierarchical Bayesian Model

Hierarchical Bayesian Model, complex but flexible, has been well recognized as a powerful mean to estimate spatial and temporal effect. The prior information included in hierarchical model is generally the intrinsic constructional information and the inferred information of parameters, which can produce accurate and sound analysis, especially with large samples. Besides, the hierarchical construction simplifies the explanation and calculation of the model, based on which Gibbs sampling can be conducted.

In this study, we assumed that the annual number of typhoid fever cases presented a Possion distribution as the incidences were at low level of hundred thousandth. The first formula was as follows:

$$\mathrm{E}(y_{\mathrm{it}}) = \lambda_{\mathrm{it}} = \mathrm{e}_{\mathrm{it}}\theta_{\mathrm{it}}$$

y_{it} is the observed number of cases at spot i in year t; e_{it} is the expected number of cases; θ_{it} is their ratio (the relative risk).

The logit connection of θ_{it} was employed to construct the second formula. Bayesian models were as follows, one containing independent spatial and temporal effect, and the other containing interactive spatiotemporal effect.

Model 1:

$$\log(\theta_{\mathrm{it}}) = \mu_0 + \mu_{\mathrm{i}} + \nu_{\mathrm{i}}$$

μ_0 is the intercept; μ_{i} is independent spatial effect and assumed to obey conditional autoregressive process (CAR); ν_{i} is the independent temporal effect and assumed to obey first-order autoregressive process(AR1).

Model 2:

$$\log(\theta_{\mathrm{it}}) = \mu_0 + \mu_{\mathrm{it}}$$

μ_{it} is the interactive effect of spot i in year t and assumed to obey CAR.

Deviance information criterion (DIC) was used to compare fitness of the two models, and the better one with low DIC was chosen. and Markov chain Monte Carlo (MCMC) algorithm to estimate parameters (μ_{i}, ν_{i}, and μ_{it}). We ran the sample for 20000 iterations after the model was stable.

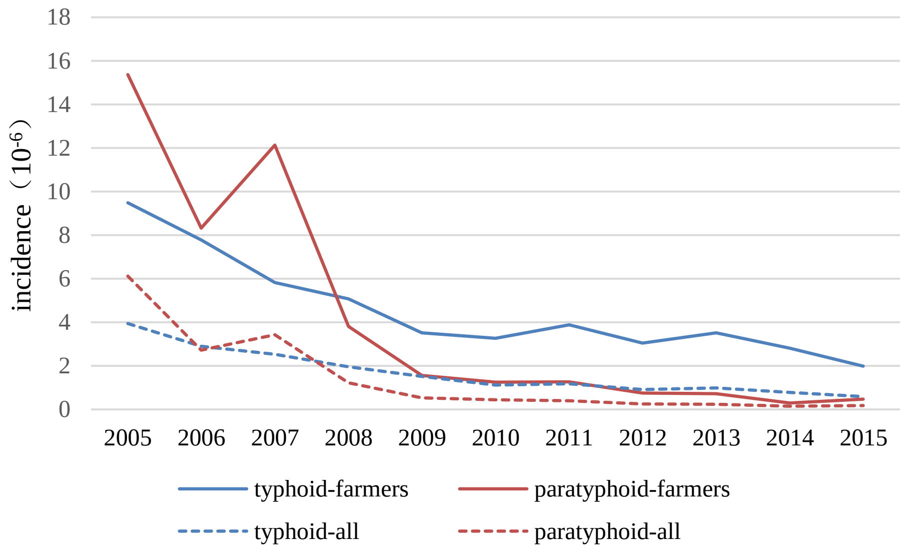

Figure S1 Typhoid and paratyphoid incidences among farmers and overall population

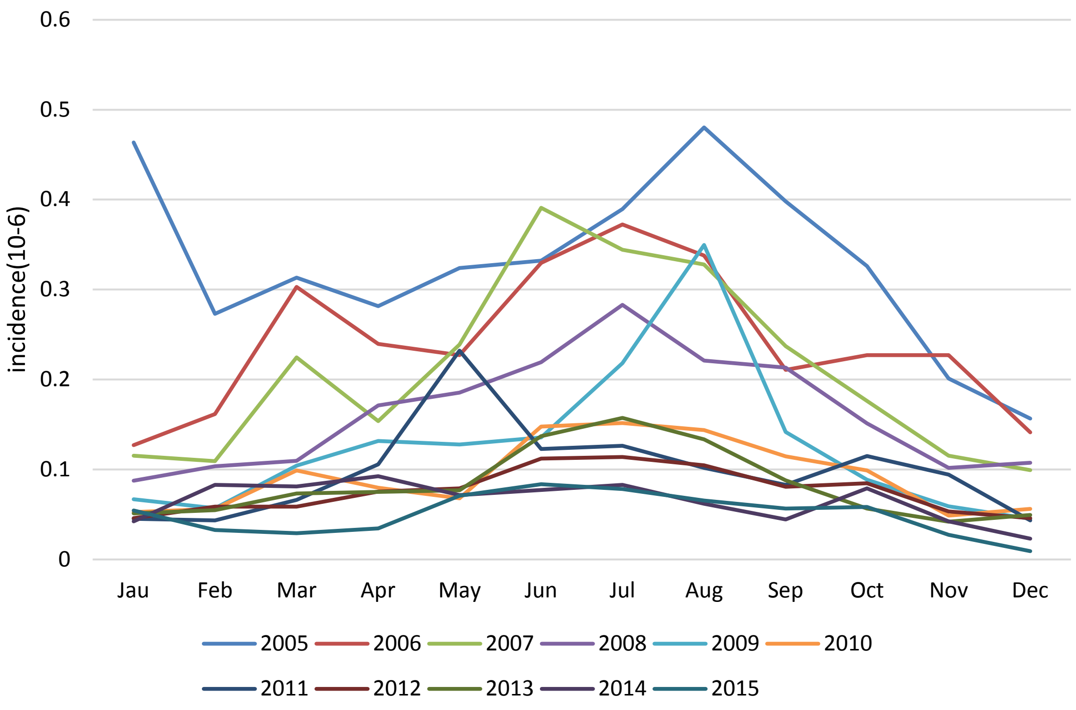

Figure S2 Monthly incidences of typhoid in Zhejiang Province from 2005 to 2015

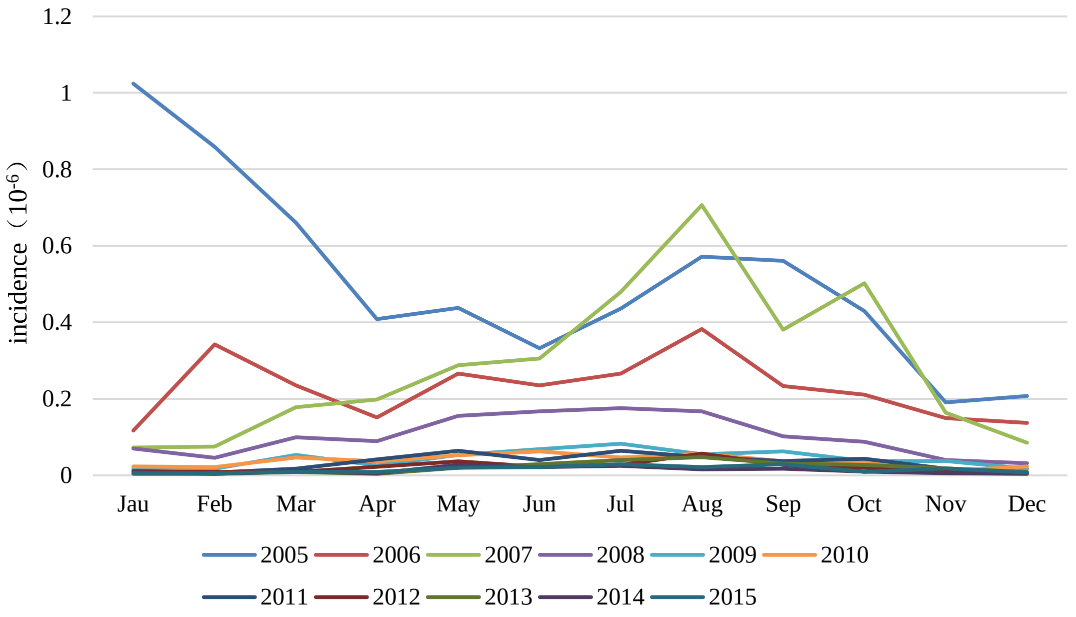

Figure S3 Monthly incidences of paratyphoid in Zhejiang Province from 2005 to 2015

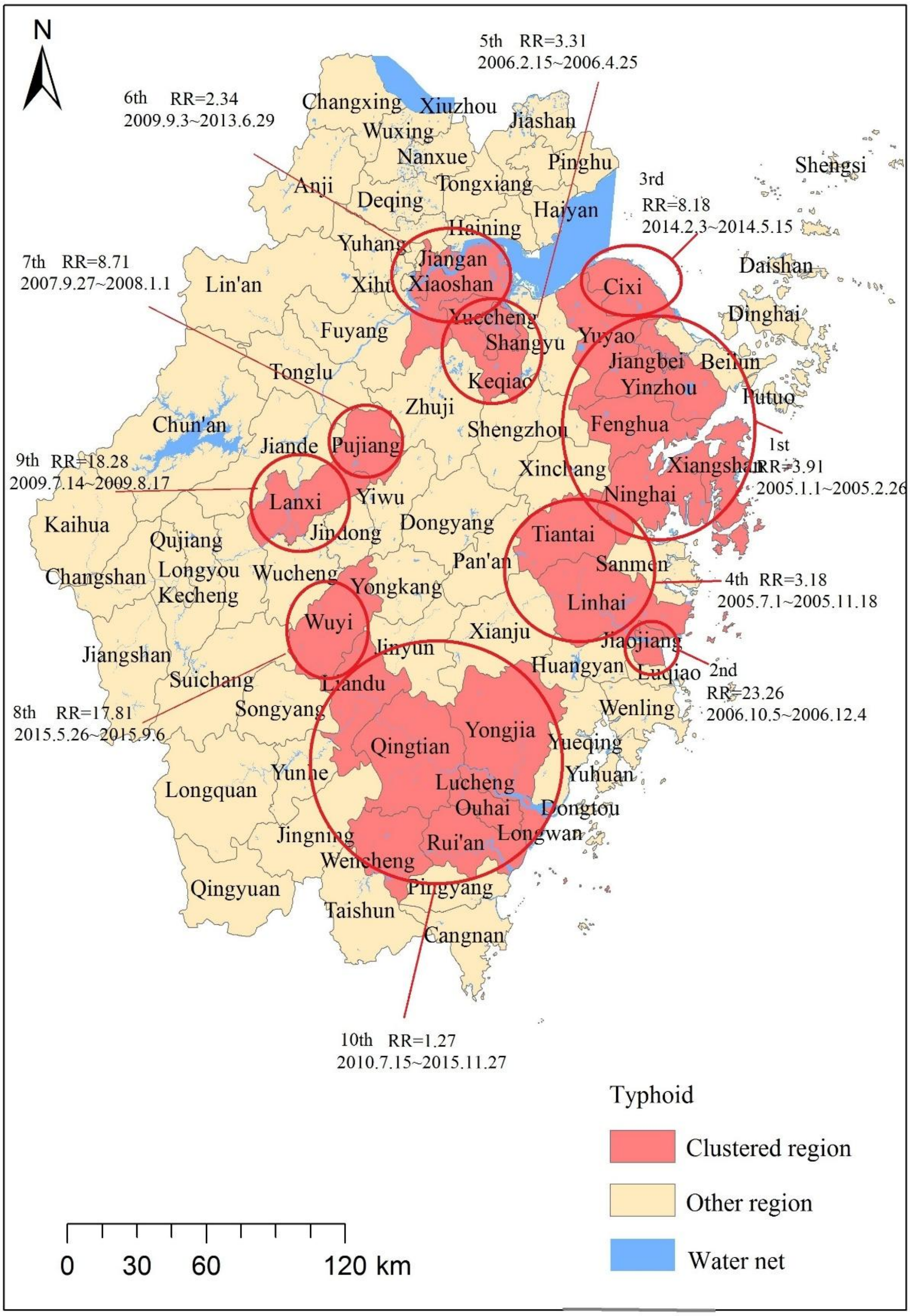

Figure S4. Spatio-temporal scan of typhoid fever in Zhejiang Province from 2005 to 2015. This map was created by ArcGIS software (version 10.1, ESRI Inc.; Redlands, CA, USA; homepage: https://www.esri.com/).

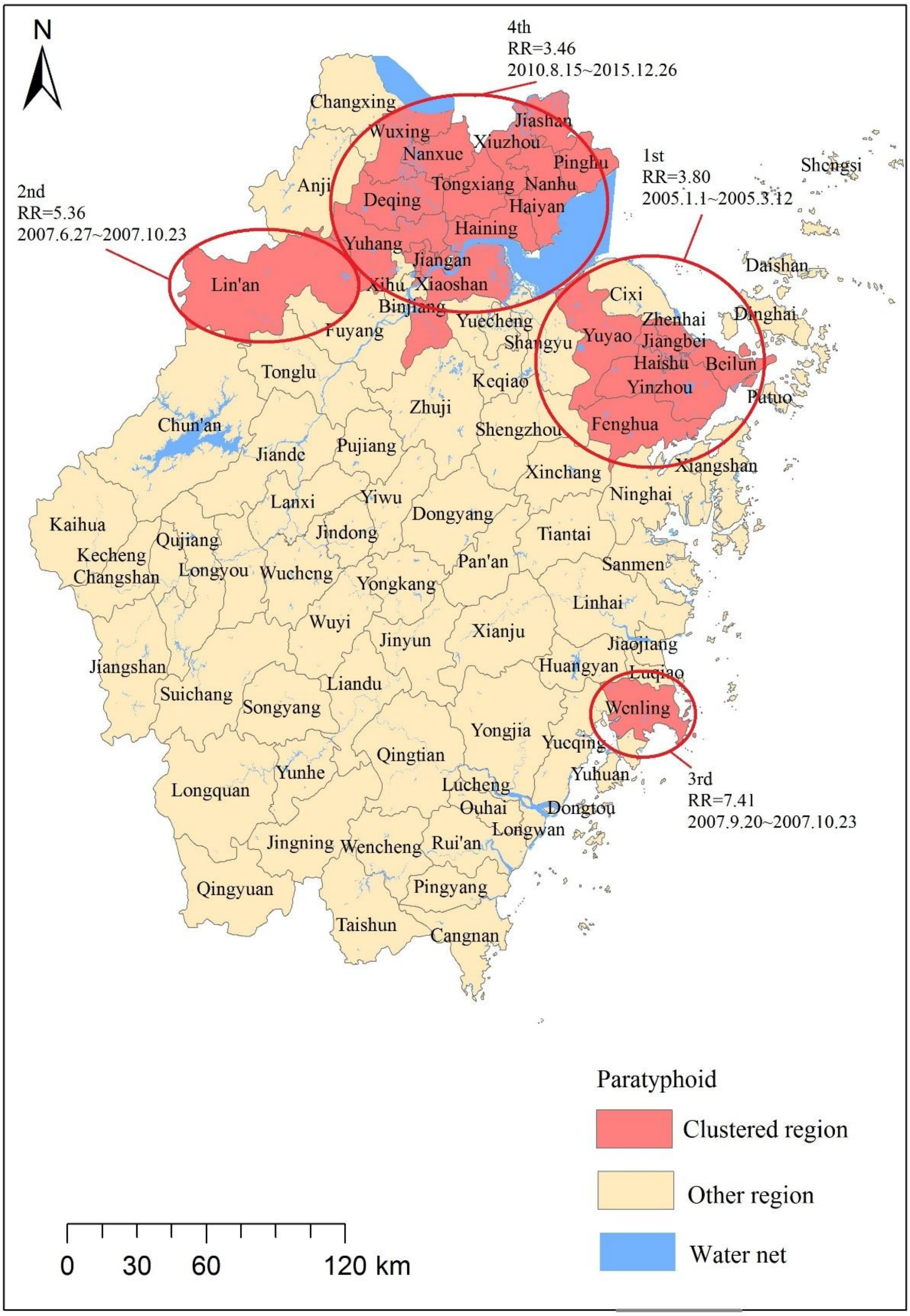

Figure S5. Spatio-temporal scan of paratyphoid fever in Zhejiang Province from 2005 to 2015. This map was created by ArcGIS software (version 10.1, ESRI Inc.; Redlands, CA, USA; homepage: https://www.esri.com/).

Table S1. Local autocorrelation analysis of typhoid in Zhejiang Province from 2005 to 2015

Year	District	Prefecture	Incidence	CoType	LMi*P*	Year	District	Prefecture	Incidence	CoType	LMi*P*
2005	Haishu	Ningbo	10.262	HH	0.0000	2010	Lucheng	Wenzhou	6.337	HH	0.0000
2005	Jiangdong	Ningbo	19.355	HH	0.0000	2010	Longwan	Wenzhou	4.135	HH	0.0000
2005	Jiangbei	Ningbo	9.710	HH	0.0000	2010	Ouhai	Wenzhou	1.990	HH	0.0000
2005	Beilun	Ningbo	5.891	HH	0.0172	2010	Dongtou	Wenzhou	2.794	HH	0.0013
2005	Zhenhai	Ningbo	7.129	HH	0.0000	2010	Yongjia	Wenzhou	2.027	HH	0.0053
2005	Yinzhou	Ningbo	13.107	HH	0.0000	2010	Yuecheng	Shaoxing	3.126	HH	0.0000
2005	Yuyao	Ningbo	9.844	HH	0.0000	2010	Keqiao	Shaoxing	2.578	HH	0.0013
2005	Cixi	Ningbo	9.056	HH	0.0000	2010	Shangyu	Shaoxing	1.760	HH	0.0024
2005	Ouhai	Wenzhou	7.002	HH	0.0145	2010	Qingtian	Lishui	0.247	LH	0.0366
2005	Yuecheng	Shaoxing	19.492	HH	0.0000	2011	Zhenhai	Ningbo	4.460	HH	0.0000
2005	Keqiao	Shaoxing	10.462	HH	0.0000	2011	Yuyao	Ningbo	3.266	HH	0.0000
2006	Linhai	Taizhou	5.523	HH	0.0122	2011	Cixi	Ningbo	1.884	HH	0.0021
2006	Lucheng	Wenzhou	11.434	HH	0.0246	2011	Lucheng	Wenzhou	4.796	HH	0.0000
2006	Yuecheng	Shaoxing	19.098	HH	0.0000	2011	Longwan	Wenzhou	3.135	HH	0.0000
2006	Keqiao	Shaoxing	9.912	HH	0.0000	2011	Ouhai	Wenzhou	3.153	HH	0.0000
2006	Shangyu	Shaoxing	3.672	HH	0.0335	2011	Yuecheng	Shaoxing	4.071	HH	0.0000
2006	Jiaojiang	Taizhou	11.051	HH	0.0000	2011	Shangyu	Shaoxing	3.638	HH	0.0000
2006	Huangyan	Taizhou	5.567	HH	0.0299	2012	Shangcheng	Hangzhou	3.307	HL	0.0275
2006	Luqiao	Taizhou	5.042	HH	0.0092	2012	Jiangbei	Ningbo	1.663	HH	0.0063
2007	Zhenhai	Ningbo	6.270	HH	0.0065	2012	Beilun	Ningbo	1.966	HH	0.0261
2007	Yuyao	Ningbo	5.928	HH	0.0001	2012	Zhenhai	Ningbo	5.997	HH	0.0000
2007	Cixi	Ningbo	5.331	HH	0.0079	2012	Yuyao	Ningbo	2.074	HH	0.0001

2007	Lucheng	Wenzhou	11.056	HH	0.0000	2012	Cixi	Ningbo	1.636	HH	0.0062
2007	Longwan	Wenzhou	13.122	HH	0.0000	2012	Lucheng	Wenzhou	4.645	HH	0.0453
2007	Ouhai	Wenzhou	4.098	HH	0.0006	2012	Shangyu	Shaoxing	2.997	HH	0.0018
2007	Yongjia	Wenzhou	5.406	HH	0.0276	2012	Yongjia	Wenzhou	2.650	HH	0.0354
2007	Yuecheng	Shaoxing	9.602	HH	0.0103	2013	Jiangbei	Ningbo	3.043	HH	0.0000
2007	Keqiao	Shaoxing	5.486	HH	0.0110	2013	Zhenhai	Ningbo	7.659	HH	0.0000
2008	Jiangbei	Ningbo	5.831	HH	0.0000	2013	Yinzhou	Ningbo	2.277	HH	0.0011
2008	Zhenhai	Ningbo	4.120	HH	0.0007	2013	Yuyao	Ningbo	3.154	HH	0.0000
2008	Yuyao	Ningbo	4.870	HH	0.0000	2013	Cixi	Ningbo	4.150	HH	0.0000
2008	Cixi	Ningbo	3.613	HH	0.0026	2013	Lucheng	Wenzhou	3.787	HH	0.0001
2008	Lucheng	Wenzhou	8.381	HH	0.0000	2013	Ouhai	Wenzhou	1.908	HH	0.0054
2008	Longwan	Wenzhou	8.268	HH	0.0000	2013	Yongjia	Wenzhou	2.268	HH	0.0246
2008	Ouhai	Wenzhou	2.859	HH	0.0141	2013	Shangyu	Shaoxing	2.467	HH	0.0023
2008	Yuecheng	Shaoxing	10.909	HH	0.0000	2014	Jiangbei	Ningbo	2.202	HH	0.0000
2008	Keqiao	Shaoxing	4.710	HH	0.0000	2014	Zhenhai	Ningbo	5.702	HH	0.0000
2009	Haishu	Ningbo	2.376	HH	0.0067	2014	Cixi	Ningbo	6.867	HH	0.0000
2009	Jiangdong	Ningbo	7.043	HH	0.0000	2014	Lucheng	Wenzhou	4.311	HH	0.0002
2009	Yuyao	Ningbo	4.278	HH	0.0000	2014	Yongjia	Wenzhou	1.893	HH	0.0060
2009	Yinzhou	Ningbo	4.756	HH	0.0000	2015	Jiangdong	Ningbo	1.082	HH	0.0260
2009	Lucheng	Wenzhou	7.643	HH	0.0000	2015	Jiangbei	Ningbo	1.647	HH	0.0000
2009	Longwan	Wenzhou	5.104	HH	0.0000	2015	Zhenhai	Ningbo	4.978	HH	0.0000
2009	Ouhai	Wenzhou	2.814	HH	0.0006	2015	Cixi	Ningbo	1.357	HH	0.0000
2009	Rui'an	Wenzhou	0.429	LH	0.0450	2015	Lucheng	Wenzhou	2.074	HH	0.0081
2009	Yuecheng	Shaoxing	6.318	HH	0.0023	2015	Yongjia	Wenzhou	2.266	HH	0.0331
2009	Keqiao	Shaoxing	2.720	HH	0.0282	2015	Pingyang	Wenzhou	1.304	HH	0.0024
2009	Shangyu	Shaoxing	2.412	HH	0.0343	2015	Cangnan	Wenzhou	1.929	HH	0.0141

2009	Kaihua	Quzhou	5.134	HL	0.0048	2015	Wencheng	Wenzhou	2.342	HH	0.0250
2010	Zhenhai	Ningbo	3.025	HH	0.0157	2015	Rui'an	Wenzhou	1.116	HH	0.0225
2010	Yuyao	Ningbo	3.178	HH	0.0000	2015	Wuyi	Jinhua	3.115	HL	0.0000
2010	Cixi	Ningbo	2.867	HH	0.0000						

Table S2. Local autocorrelation analysis of paratyphoid in Zhejiang Province from 2005 to 2015

Year	District	Prefecture	incidence	CoType	LMi*P*	Year	District	Prefecture	incidence	CoType	LMi*P*
2005	Xihu	Hangzhou	27.280	HL	0.0068	2012	Yueqing	Wenzhou	0.000	LH	0.0151
2005	Haishu	Ningbo	45.577	HH	0.0000	2012	Yuhuan	Taizhou	2.107	HL	0.0000
2005	Jiangdong	Ningbo	36.519	HH	0.0000	2012	Jingning Shezu Zizhixian	Lishui	0.928	HL	0.0140
2005	Jiangbei	Ningbo	31.462	HH	0.0000	2013	Xihu	Hangzhou	0.755	HH	0.0000
2005	Beilun	Ningbo	54.897	HH	0.0000	2013	Shangcheng	Hangzhou	0.599	HH	0.0000
2005	Zhenhai	Ningbo	26.536	HH	0.0000	2013	Gongshu	Hangzhou	0.563	HH	0.0029
2005	Yinzhou	Ningbo	21.163	HH	0.0000	2013	Binjiang	Hangzhou	0.651	HH	0.0000
2005	Fenghua	Ningbo	35.730	HH	0.0000	2013	Xiaoshan	Hangzhou	0.114	LH	0.0377
2006	Lin'an	Hangzhou	32.039	HL	0.0157	2013	Yuhang	Hangzhou	0.529	HH	0.0013
2006	Yuhuan	Taizhou	25.085	HH	0.0146	2013	Xiasha	Hangzhou	2.221	HH	0.0000
2006	Wenling	Taizhou	8.767	HH	0.0034	2013	Jiangan	Hangzhou	0.756	HH	0.0000
2007	Lin'an	Hangzhou	16.322	HL	0.0394	2013	Wuyi	Jinhua	1.139	HL	0.0115
2007	Yuhuan	Taizhou	102.333	HH	0.0000	2013	Deqing	Huzhou	0.000	LH	0.0015
2008	Lin'an	Hangzhou	14.217	HL	0.0030	2014	Jiangan	Hangzhou	0.758	HH	0.0002
2008	Tonglu	Hangzhou	0.000	LH	0.0282	2014	Xiasha	Hangzhou	1.621	HH	0.0014

2008	Pujiang	Jinhua	13.396	HL	0.0000	2014	Dongtou	Wenzhou	0.000	LH	0.0418
2009	Jiangdong	Ningbo	1.921	HH	0.0267	2014	Yueqing	Wenzhou	0.000	LH	0.0151
2009	Wuyi	Jinhua	2.452	HL	0.0003	2014	Yuhuan	Taizhou	2.107	HL	0.0000
2009	Pujiang	Jinhua	4.156	HL	0.0016	2014	Jingning Shezu Zizhixian	Lishui	0.928	HL	0.0140
2010	Jiangbei	Ningbo	1.344	HH	0.0002	2015	Xihu Qu	Hangzhou	0.497	HH	0.0000
2010	Zhenhai	Ningbo	2.689	HH	0.0000	2015	Xiacheng	Hangzhou	0.969	HH	0.0000
2010	Yueqing	Wenzhou	0.000	LH	0.0382	2015	Shangcheng	Hangzhou	0.592	HH	0.0000
2010	Dongtou	Wenzhou	0.000	LH	0.0075	2015	Gongshu	Hangzhou	0.369	HH	0.0032
2010	Yuhuan	Taizhou	2.692	HL	0.0003	2015	Binjiang	Hangzhou	1.598	HH	0.0000
2011	Dongtou	Wenzhou	0.000	LH	0.0156	2015	Xiaoshan	Hangzhou	0.056	LH	0.0198
2011	Pujiang	Jinhua	2.811	HL	0.0097	2015	Xiasha	Hangzhou	0.631	HH	0.0003
2011	Yuhuan	Taizhou	5.790	HL	0.0000	2015	Jiangan	Hangzhou	0.000	LH	0.0024
2011	Yunhe	Lishui	1.767	HH	0.0012	2015	Haining	Jiaxing	0.611	HH	0.0000
2012	Jiangan	Hangzhou	0.758	HH	0.0002	2015	Deqing	Huzhou	0.000	LH	0.0196
2012	Xiasha	Hangzhou	1.621	HH	0.0014	2015	Yuhuan	Taizhou	0.646	HL	0.0407
2012	Dongtou	Wenzhou	0.000	LH	0.0418	2015	Huangyan	Taizhou	0.777	HL	0.0037
